# Pulpal blood flow changes and pain scores related to using Superelastic 0.018-inch Nickel Titanium as the first orthodontic alignment archwire: a prospective clinical trial

**DOI:** 10.1590/1678-7757-2021-0089

**Published:** 2021-10-04

**Authors:** Elham S. Abu ALHAIJA, Ahmad Y. SHAHIN, Serene A. BADRAN, Saba O. DAHER, Hasan O. DAHER

**Affiliations:** 1 Qatar University College of Dental Medicine Doha Qatar Qatar University, College of Dental Medicine, QU Health, Doha, Qatar.; 2 Jordan University of Science and Technology Faculty of Dentistry Department of Preventive Dentistry Irbid Jordan Jordan University of Science and Technology, Faculty of Dentistry, Department of Preventive Dentistry Division of Orthodontics, Irbid-Jordan.; 3 University of Jordan Faculty of Dentistry Department of Pediatric Dentistry and Orthodontics Amman Jordan University of Jordan, Faculty of Dentistry, Department of Pediatric Dentistry and Orthodontics, Amman-Jordan.; 4 Jordan University of Science and Technology Faculty of Medicine Irbid Jordan Jordan University of Science and Technology, Faculty of Medicine, Irbid-Jordan.

**Keywords:** Fixed appliance, Laser Doppler Flowmeter, Pain, Archwire, Alignment

## Abstract

**Background:**

Optimal orthodontic force results in maximum rate of tooth movement without tissue damage. Even though starting orthodontic treatment with a thicker archwire may shorten treatment duration, the evidence on the effect of using 0.018-inch NiTi as the first alignment archwire on pulpal blood flow (PBF) status is still scarce.

**Objectives:**

to record PBF changes and pain scores associated with using 0.018-inch NiTi as the first alignment archwire during fixed orthodontic treatment.

**Methodology:**

Patients were selected from subjects attending postgraduate orthodontic teaching clinics at Jordan University of Science and Technology. In total, forty healthy patients who exhibited mild lower arch crowding were included. A split-mouth trial design was used. Each patient received two archwire sizes at one time joined in the midline by crimpable hook and applied in the lower arch. Patients were assigned into one of two groups based on archwire sizes used. Group 1: 0.014-inch and 0.018-inch NiTi (Six males, 14 females aged 19.4±1.33 years) and Group 2: 0.016-inch and 0.018-inch NiTi (Seven males, 13 females aged 19.6±1.45 years). The archwire size group was randomly allocated with a 1:1 allocation ratio. A Laser Doppler Flowmeter was used to measure PBF at different time intervals (T0-T5). Pain scores were recorded using a visual analogue scale (VAS). A repeated measures ANOVA and a post-hoc Bonferroni comparison tests were conducted to examine differences at the different time points before and during orthodontic alignment.

**Results:**

For all studied archwire sizes, PBF decreased 20 minutes after their placement. Most PBF changes occurred within 24hours and continued to decrease until 72 hours after archwire placement where the maximum reduction was reached. Eventually, normal values were reverted within 1 month. PBF changes were similar between all alignment – groups.

**Conclusions:**

Initial orthodontic alignment with 0.018-inch NiTi does not cause irreversible changes to pulpal vasculature or produces higher pain scores.

## Introduction

The initial alignment archwire is the first to be inserted into the orthodontic brackets at the start of orthodontic treatment. The archwire is used for the correction of tooth malposition and displacement. Superelastic nickel-titanium (NiTi) wires have been widely used as initial alignment archwires due to their unique characteristics of superelasticity and shape memory. They are known for applying the same amount of force regardless of the amount of their deflection and are especially valuable where large deflections are needed to align malpositioned teeth.

Subsequent to orthodontic force application, the most important pulpal changes that may occur as reported by histologic studies include, decrease of pulp tissue respiration, vascular changes, hemorrhage, fibro-hyalinosis and necrosis.^1-2^ Both mechanical pressure on the periodontal ligament (PDL) and changes in blood flow result in release of several neurotransmitters, cytokines and growth factors involved in tooth movement.^3^

The impact of orthodontic force on human dental pulp was systematically reviewed by Javed, et al. ^4^ (2014). The study investigates the effect of orthodontic forces on pulpal blood flow (PBF) and the pulpal cellular reactions to orthodontic forces. The authors, however, found no scientific evidence of any association between orthodontic forces and human dental pulp irreversible changes.

The magnitude of force and stress produced by initial alignment archwire were previously investigated. Badran, et al.^5^ (2003) used a photo-elastic model to study the stresses transmitted to the roots of teeth by various alignment archwires. They reported higher stress values for non-elastic NiTi archwires compared to the super-elastic archwires. The shear stresses continued to increase 24 hours after initial engagement of archwires. Francisconi, et al.^6^ (2016) reported that the deactivation forces delivered by non-superelastic NiTi archwires were greater than those produced by their superelastic counterparts.

During orthodontic treatment, mild forces are preferable to minimize damage to teeth and surrounding structures, and produce favorable tooth displacement. However, von Böhl, et al.^7^ (2004) stated that the rate of tooth movement is determined by the degree of PDL hyalinization and the rate of removal of necrotic tissue in response to heavy orthodontic force and not on force magnitude. Based on this, Krishnan and Davidovitch^3^ (2006) defined optimal orthodontic force as “a force of certain magnitude that leads to the maximum rate of tooth movement without tissue damage and maximum patient comfort” suggesting that a similar rate of tooth movement would be achieved with a wide range of force magnitude.^3^

In a systematic review to assess the effects of initial alignment archwires during fixed orthodontic treatment on speed of alignment or pain, Jian, et al.^8^ (2013) did not find any evidence that any initial archwire is better or worse than another, suggesting that force magnitude has a lesser role in the rate of orthodontic movement. On the other hand, Luppanapornlarp, et al.^9^ (2010) reported that at 24 hours after force application, the mean pain scores from the 150 g force was greater than the test that performed 50 g.

Pulpal responses to orthodontic forces where different magnitude and duration of orthodontic forces were applied have already been investigated.^10-17^ Although starting orthodontic treatment with a thicker archwire may produce optimal forces and shorten treatment duration, the effect of using 0.018-inch superelastic NiTi as initial alignment archwire on PBF status has not been studied before. To our knowledge, this is the first study to compare PBF between different initial superelastic NiTi archwires sizes during alignment and leveling phase of orthodontic treatment. Our study aimed to:

investigate changes in PBF associated with orthodontic forces during leveling and alignment using superelastic 0.018-inch NiTi as an initial arch wire at 20 minutes, 24 hours, 72 hours, 1 week, 1 month time points.

compare PBF of the above archwire with the more commonly used 0.014-inch NiTi and 0.016-inch NiTi archwires at the same time intervals

investigate pain score related to orthodontic alignment with 0.018-inch NiTi archwire.

compare pain scores related to 0.018-inch NiTi archwire with the more commonly used 0.014-inch NiTi and 0.016-inch NiTi archwires

### Null Hypothesis

PBF changes after initial alignment with 0.014-inch, 0.016-inch or 0.018-inch NiTi are different.

## Methodology

This study was performed from September 2018 to December 2019 after institutional review board approval at Jordan University of Science and Technology/ King Abdullah II University Hospital (IRB No. 35/111/2017). It was registered at ClinicalTrial.gov (NCT04378205).

**Figure 1:**
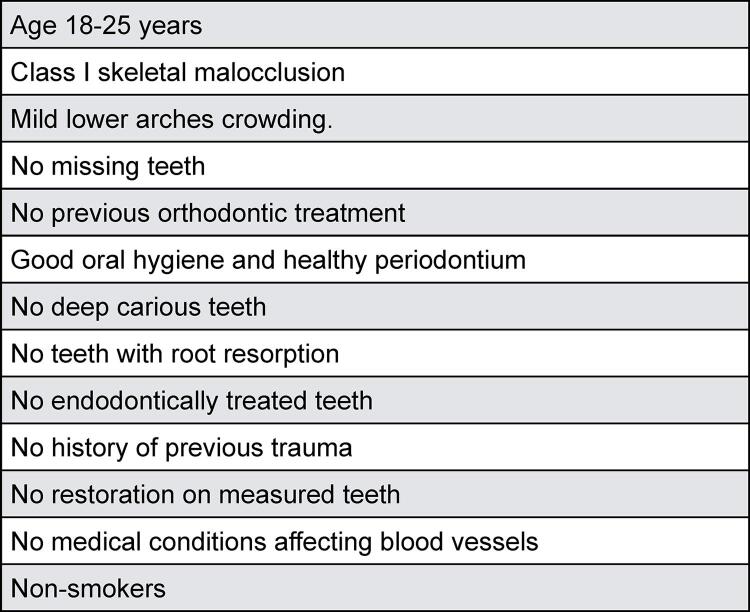
Inclusion criteria

The patients selected for this study attended the postgraduate orthodontic teaching clinics/ Jordan University of Science and Technology. Subjects were chosen based on the inclusion criteria shown in Figure 1. A consent form was signed by all subjects included after explained the study purpose.

The G*Power 3.1.9 program was used to determine sample size. Assuming a small effect size (0.25), at an alpha level (0.05) and a desired power (1 – β) of 0.95, a total sample size of 28 subjects was generated. After adding 15% overall attrition rate, 32 patients (16 patient/group) should be included.

Following patients’ recruitment, the archwire size groups were randomly allocated (permuted block size of 2) with a 1:1 allocation ratio. Afterwards, the random allocation of archwire size to either the right or left sides were concealed in envelopes and each subject was inquired to select one. After initiation, trial design was not changed throughout the study. The patient was blinded to the intervention used, but it was not possible to blind the clinician during treatment. However, the measurements of the PBF were performed by a research assistant (S.D), who was blinded to the type of the intervention used and side.

In total, forty healthy patients (28 females and 12 males) aged from 18 to 25 years (19.5±1.39 years) that required orthodontic treatment with fixed appliance were selected to participate in the study. All included subjects were diagnosed with Class I malocclusion (ANB 3.96±0.89°) with mild lower arch crowding (4.19±0.86 mm) and were treated with fixed appliance as non-extraction plan. Teeth displacement averaged was 1.97±٠.٣٠ mm. Pre-treatment records were taken for all subjects (orthopantomogram, lateral cephalogram and study casts).

A split mouth trial design was used. One orthodontic resident (A.S.) performed orthodontic treatment for all patients using pre-adjusted edgewise-fixed appliance (3M Gemini Unitek brackets; 0.022-inch Roth prescription). Each patient received 2 alignment archwire sizes (Nitinol SuperElastic 3M, Unitek) at one time joined in the midline by crimpable hook and applied in the lower arch. Patients were followed monthly.

Patients were subdivided into 2 groups:

Group (1): 0.014-inch NiTi - 0.018-inch NiTi archwires for initial alignment. Right or left sides of the patients (six males, 14 females aged, 19.4±1.33 years) were included.

Group (2): 0.016-inch NiTi- 0.018-inch NiTi archwires for initial alignment. Right or left sides of the patients (seven males, 13 females, aged 19.6±1.45 years) were included.

**Figure 2:**
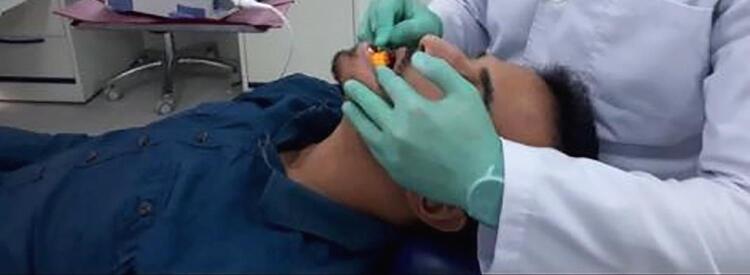
PBF measurement using LDF

PBF measurements were taken using the Laser Doppler Flowmeter (LDF) (Moor lab, Moor instruments, UK) calibrated following the manufacturer’s instructions. With a wavelength of 780 nm and a dental probe MP 13 (Moor instruments, UK; 2 fibers, 0.25 mm diameter, centers 0.5 mm spaced a part), all measurements were performed by the one researcher in a standard position (Figure 2). Room temperature was kept in the range from 20°C to 25°C. Patients were provided with a 15 minutes’ rest before starting PBF measurements.

Before recording PBF measurement, a silicone splint was constructed for each patient to ensure dental probe stability during PBF measurements. Holes were made with a drill of 1.5 mm diameter below the brackets’ imprints in the mold, to allow the probe to touch the teeth. This will ensure registering PBD on the same position at the different time points. The silicone splints were manufactured to extend over the attached gingiva. The retentive areas of the brackets and the labial gingiva were covered with utility wax. To help the isolation of the teeth during measurements and minimize the contamination of the blood-flow signals from adjacent tissues^18^, four cotton rolls were applied in the gingival sulcus, keeping the lower lip and the cheeks away.

PBF was recorded and expressed in perfusion units (PU) at the following time points:

Before brackets bond up in both groups. These values were considered as the basal blood flow (T0).

Twenty minutes after placement of the initial wires (T1).

Twenty-four hours after placement of the wires (T2).

Seventy-two hours after placement of the wires (T3).

One week after placement of the wires (T4).

One month after placement of the wires (T5).

Data were taken for each tooth until 2 minutes of stable PBF value was registered on the flowmeter screen. Once all the PBF measurements were recorded (30-45min/patient), the silicone splint was removed for that time point session recording.

Pain was assessed in each subject by measuring both pain intensity and interference using Visual Analog Scale (VAS) (0 = no pain and 10 = worst pain). The participants filled out a questionnaire to assess the pain intensity and interference after archwire insertion at 1, 2, and 7 days of intervention. The patients were asked to provide their subjective answers regarding pain during a day and the dose and time of using analgesics. The duration of this study was 1 month after fitting the initial alignment archwires.

### Interim analyses and stopping guidelines

Not applicable

### Statistical analysis

Data analysis was performed with SPSS (22.0, SPSS Inc., NY, USA). Descriptive statistics for PBF and pain scores at the different time intervals were estimated. Within-subject’s ANOVA repeated measures analysis of variance and *post-hoc* Bonferroni comparison tests were applied to examine differences in PBF and pain scores at the different time points. Paired *t*-test was applied to detect differences between the right and left sides of the patients. A threshold probability value of p≤0.05 was predetermined to indicate statistical significance.

### Method error

Measurement error using Dahlberg formula and Houston’s coefficient of reliability were estimated. Dahlberg error was 0.6 PU and the coefficient of reliability was 88% indicating substantial agreement.

## Results

Tables 1-4 shows the means, standard deviations (SD), differences (Diff.) between the means and p-values for PBF from baseline (T0) to (T5) for each measured tooth for each archwire size.

**Table 1:** Means and SD for PBF (PU) from baseline (T0) to (T5) for each measured tooth/quadrant for each studied archwire

Tooth	T0	T1	T2	T3	T4	T5
	Mean (SD)	Mean (SD)	Mean (SD)	Mean (SD)	Mean (SD)	Mean (SD)
**SuperElastic 0.014-inch NiTi Archwire (n=20)**						
1^st^ molar	7.87(0.38)	6.894 (0.30)	5.18 (0.34)	3.80 (0.38)	5.85 (0.78)	7.83 (0.28)
2^nd^ premolar	7.84 (0.36)	6.92 (0.34)	5.26 (0.34)	3.81 (0.34)	5.80 (0.78)	7.76 (0.17)
1^st^ premolar	7.97 (0.37)	7.06 (0.29)	5.14 (0.31)	3.66 (0.35)	5.77 (0.78)	7.84 (0.28)
Canine	7.91 (0.34)	6.97 (0.35)	5.17 (0.37)	3.69 (0.37)	5.74 (0.71)	7.96 (0.24)
Lateral Incisor	7.93 (0.40)	6.96 (0.40)	5.24 (0.39)	3.73 (0.33)	5.79 ( 0.63)	7.75 (0.27)
Central Incisor	7.89 (0.31)	6.99 (0.31)	5.25 (0.32)	3.72 (0.36)	5.80 (0.81)	7.74 (0.32)
**SuperElastic 0.016-inch NiTi Archwire (n=20)**						
1^st^ molar	7.98 (0.51)	7.12 (0.38)	5.15 (0.35)	3.73 ( 0.31)	5.59 (0.55	7.57 (0.65)
2^nd^ premolar	7.92 (0.38)	7.10 (0.44)	5.18 (0.39)	3.82 (0.36)	5.61 (0.63)	7.40 (0.65)
1^st^ premolar	7.85 (0.45)	6.89 (0.40)	5.28 (0.32)	3.79 (0.32)	5.57 (0.54)	7.51 (0.59)
Canine	7.93 (0.33)	6.95 (0.44)	5.17 (0.33)	3.68 (0.28)	5.58 (0.58)	7.48 (0.48)
Lateral Incisor	7.82 (0.37)	6.88 (0.42)	5.10 (0.25)	3.79 (0.32)	5.43 ( 0.38)	7.38 (0.55)
Central Incisor	7.92 (0.30)	7.19 (0.31)	5.26 (0.36)	3.73 (0.31)	5.53 (0.55)	7.38 (0.56)
**SuperElastic 0.018-inch NiTi Archwire (n=40)**						
1^st^ molar	7.96 (0.46)	7.03 (0.43)	5.24 (0.38)	3.75 (0.28)	5.58 (0.51)	7.46 (0.65)
2^nd^ premolar	7.86 (0.40)	7.00 (0.42)	5.23 (0.32)	3.78 (0.35)	5.60 (0.53)	7.37 (0.63)
1^st^ premolar	7.87 (0.43)	6.89 (0.41)	5.29 (0.29)	3.74 (0.30)	5.56 (0.53)	7.44 (0.55)
Canine	7.90 (0.37)	6.92 (0.44)	5.20 (0.31)	3.73 (0.30)	5.59 (0.51)	7.43 (0.52)
Lateral Incisor	7.80 (0.38)	6.83 (0.39)	5.19 (0.29)	3.74 (0.34)	5.50 (0.44)	7.38 (0.56)
Central Incisor	7.84 (0.33)	7.01 (0.37)	5.26 (0.32)	3.69 (0.32)	5.48 (0.55)	7.34 (0.52)

T0: Pretreatment (baseline) / T1: 20 minutes after wire placement / T2: 24 hours after wire placement/ T3: 72 hours after wire placement /T4: 1 week after wire placement / T5: 1 month after wires placement

For all archwire sizes, PBF decreased 20 minutes after their placement (p<0.001). Significant PBF reduction occurred within the first 24 hours (p<0.001) and continued to decrease until 72 hours after archwire placement, when maximum reduction was reached (p<0.001). After that, PBF started to increase back gradually. Eventually, normal values were reverted within 1 month (p>0.05).

**Figure 3:**
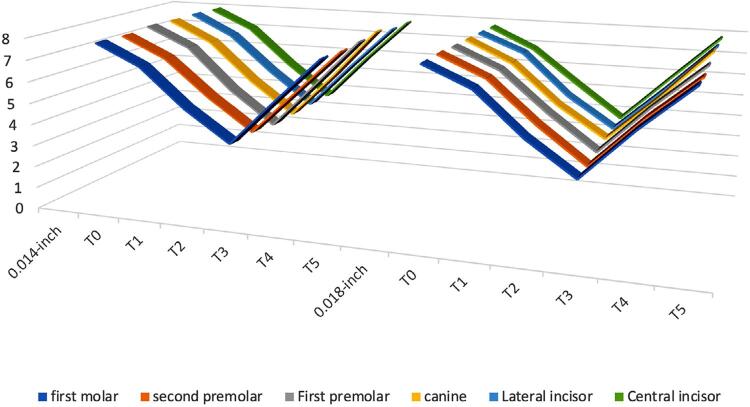
PBF changes in 0.018-inch NiTi archwire compared to 0.014-inch NiTi archwire

**Figure 4:**
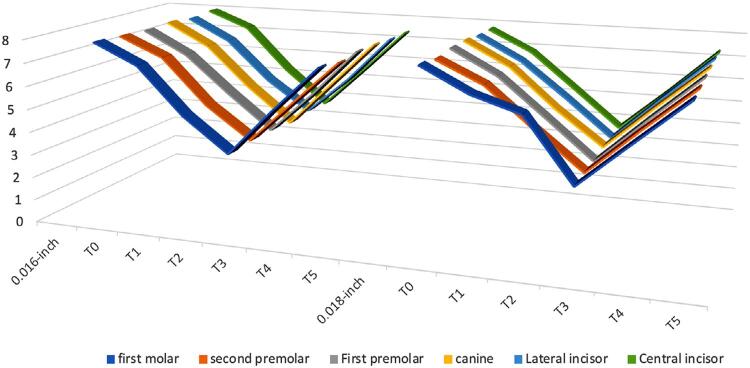
PBF changes in 0.018-inch NiTi archwire compared to 0.016-inch NiTi archwire

Table 5 and Figures 3 and 4 shows the comparison between the mean PBF between the 2 archwire sizes within the same group. PBF changes were similar between all archwire sizes (0.014-inch NiTi, 0.016-inch NiTi and 0.018-inch NiTi) (p>0.05).

**Table 5:** Means, SD, diff. between the means of the two studied groups and p-values for PBF from baseline (T0) to (T5) for each measured tooth

		0.014-inch/0.018-inch NiTi group				0.016-inch/0.018-inch NiTi group			
		0.014 NiTi	0.018 NiTi	Differences between the means	P value	0.016 NiTi	0.018NiTi	Differences between the means	P value
		Mean (SD)	Mean (SD)			Mean (SD)	Mean (SD)		
T0	first molar	7.87 (0.37)	7.93 (0.33)	-0.07	NS	7.98 (0.51)	7.93 (0.33)	0.05	NS
	Second premolar	7.84 (0.28)	7.94 (0.34)	-0.1	NS	7.91 (0.37)	7.80 (0.42)	0.12	NS
	First premolar	7.96 (0.36)	7.88 (0.39)	-0.08	NS	7.85 (0.44)	7.90 (0.42)	-0.05	NS
	Canine	7.92 (0.34)	7.85 (0.26)	0.07	NS	7.92 (0.33)	7.89 (0.42)	0.04	NS
	Lateral incisor	7.94 (0.39)	7.81 (0.37)	0.12	NS	7.82 (0.36)	7.78 (0.39)	0.04	NS
	Central incisor	7.89 (0.31)	7.77 (0.30)	0.11	NS	7.92 (0.30)	7.77 (0.35)	0.15	NS
T1	first molar	6.94 (0.29)	7.04 (0.39)	0.11	NS	7.11 (0.38)	6.94 (0.46)	0.18	NS
	Second premolar	6.92 (0.35)	7.04 (0.36)	-0.11	NS	7.10 (0.44)	6.90 (0.38)	0.2	NS
	First premolar	7.06 (0.28)	6.99 (0.29)	0.06	NS	6.88 (0.39)	6.88 (0.42)	0	NS
	Canine	6.97 (0.34)	6.91 (0.32)	0.05	NS	6.94 (0.43)	6.89 (0.46)	0.05	NS
	Lateral incisor	6.96 (0.40)	7.00 (0.31)	-0.04	NS	6.87 (0.42)	6.77 (0.35)	0.11	NS
	Central incisor	6.99 (0.31)	6.82 (0.26)	0.16	NS	7.19 (0.30)	6.83 (0.35)	0.36	***
T2	first molar	5.18 (0.34)	5.19 (0.40)	-0.01	NS	5.15 (0.34)	6.33 (0.39)	-0.18	NS
	Second premolar	5.26 (0.34)	5.23 (0.27)	0.03	NS	5.17 (0.38)	5.29 (0.22)	-0.11	NS
	First premolar	5.16 (0.30)	5.24 (0.30)	-0.08	NS	5.28 (0.31)	5.30 (0.27)	-0.03	NS
	Canine	5.17 (0.37)	5.12 (0.34)	0.04	NS	5.17 (0.33)	5.22 (0.28)	-0.05	NS
	Lateral incisor	5.24 (0.39)	5.11 (0.26)	0.12	NS	5.10 (0.25)	5.27 (0.31)	-0.17	NS
	Central incisor	5.24 (0.31)	5.25 (0.29)	-0.01	NS	5.26 (0.35)	5.26 (0.32)	0	NS
T3	first molar	3.80 (0.38)	3.74(0.22)	0.05	NS	3.72 (0.31)	3.77 (0.26)	-0.05	NS
	Second premolar	3.82 (0.34)	3.67(0.36)	0.13	NS	3.81 (0.36)	3.75 (0.35)	0.07	NS
	First premolar	3.66 (0.35)	3.72(0.28)	-0.06	NS	3.79 (0.32)	3.68 (0.27)	-0.11	NS
	Canine	3.70 (0.36)	3.77(0.26)	-0.08	NS	3.67 (0.28)	3.78 (0.31)	0.11	NS
	Lateral incisor	3.73 (0.32)	3.73(0.29)	-0.01	NS	3.79 (0.32)	3.68 (0.36)	0.06	NS
	Central incisor	3.72 (0.35)	3.73(0.27)	-0.01	NS	3.72 (0.31)	3.67 (0.32)	0	NS
T4	first molar	5.85 (0.77)	5.91 (0.85)	-0.06	NS	5.69 (0.54)	5.57 (0.49)	0.03	NS
	Second premolar	5.80 (0.77)	5.83 (0.85)	-0.03	NS	5.61 (0.63)	5.59 (0.42)	0.02	NS
	First premolar	5.73 (0.77)	5.86 (0.78)	-0.14	NS	5.56 (0.53)	5.56 (0.55)	0.01	NS
	Canine	5.74 (0.71)	5.76 (0.80)	-0.03	NS	5.57 (0.58)	5.61 (0.44)	-0.03	NS
	Lateral incisor	5.79 (0.63)	5.71 (0.64)	0.07	NS	5.43 (0.38)	5.57 (0.50)	-0.14	NS
	Central incisor	5.80 (0.81)	5.80 (0.78)	-0.01	NS	5.52 (0.55)	5.44 (0.56)	0.09	NS
T5	first molar	7.83 (0.27)	7.82 (0.37)	0.01	NS	7.56 (0.65)	7.34 (0.65)	0.22	NS
	Second premolar	7.76 (0.16)	7.71 (0.23)	0.04	NS	7.39 (0.64)	7.35 (0.62)	0.05	NS
	First premolar	7.84 (0.27)	7.77 (0.28)	0.06	NS	7.51 (0.58)	7.37 (0.53)	0.15	NS
	Canine	7.96 (0.23)	7.97 (0.27)	-0.02	NS	7.47 (0.48)	7.38 (0.57)	0.1	NS
	Lateral incisor	7.75 (0.27)	7.82 (0.26)	-0.08	NS	7.38 (0.55)	7.38 (0.59)	0	NS

NS not significant

**Figure 5:**
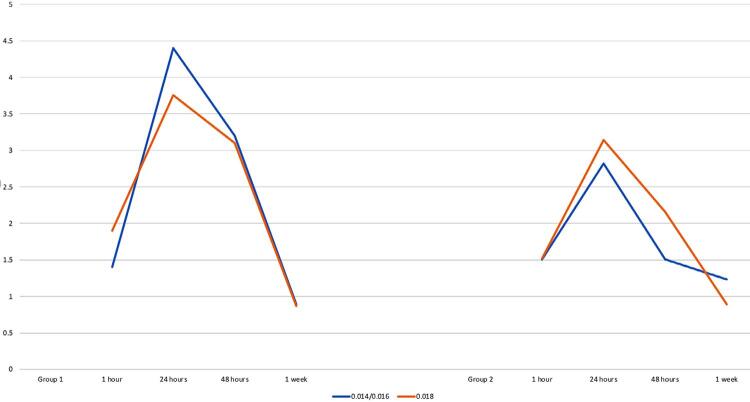
Pain scores associated with 0.018-inch NiTi archwire compared to 0.014-inch and 0.016-inch NiTi as initial alignment archwires

Tables 6 and 7 and Figure 5 shows the means, SD, Diff. between means and p-values for pain scores reported by patients from 1 hour after engagement of wires to 1 week for each archwire size. Pain increased immediately after the archwires were inserted and reached its peak after 48 hours; thereafter, pain started to decline to the pre-treatment level. Similar pain scores were recorded for the different alignment archwire sizes used (p>0.05).

**Table 6:** Means and SD for pain scores for each group and the difference between the two archwire sizes within the same group

	Group 1			Group 2		
Time	0.014-inch NiTi	0.018-inch NiTi	Diff between means	0.016-inch NiTi	0.018-inch NiTi	Diff between means
1 hour	1.40 (2.64)	1.90 (2.98)	-0.50 NS	1.51 (2.15)	1.52 (1.83)	-0.01 NS
24 hours	4.40 (3.24)	3.76 (3.23)	0.64 NS	2.82 (2.49)	3.14 (2.59)	-0.32 NS
48 hours	3.20 (2.95)	3.10 (3.09)	0.10 NS	1.51 (1.68)	2.16 (2.15)	-0.65 NS
1 week	0.89 (1.22)	0.87 (1.21)	0.02 NS	1.23 (2.57)	0.89 (2.03)	0.34 NS

NS: not significant

**Table 7:** Diff. between means and p-values for the pain scores at the different time points in each archwire size group

Tooth	Archwire size	F value	Diff	p-value	Diff	p-value	Diff	p-value	Diff	p-value	Diff	p-value	Diff	
			T1-T2		T1-T3		T1-T4		T2-T3		T2-T4		T3-T4	p-value
			Mean (SE)		Mean (SE)		Mean (SE)		Mean (SE)		Mean (SE)		Mean (SE)	
Group 1	0.014-inch	12.62	3.00 (0.80)	0.001	1.80 (0.82)	0.041	-0.52 (0.58)	0.385	-1.20 (0.64)	0.08	-3.52 (0.59)	0.001	-2.32 (0.52)	0.001
		***		***		*		NS		NS		***		***
	0.018-inch	10.69	1.86 (1.02)	0.08	1.20 (1.06)	0.27	-1.04 (0.77)	0.19	-0.66 (0.54)	0.24	-2.90 (0.55)	0.001	-2.24 (0.58)	0.001
		***		NS		NS		NS		NS		***		***
Group 2	0.016-inch	2.49	1.31 (0.61)	0.046	-0.01 (0.65)	0.99	-0.29 (0.81)	0.73	-1.31 (0.52)	0.021	-1.59 (0.78)	0.056	-0.28 (0.59)	0.63
		NS		*		NS		NS		*		NS		NS
	0.018-inch	3.57	1.63 (0.60)	0.014	0.65 (0.68)	0.34NNS	-0.63 (0.62)	0.32	-0.98 (0.52)	0.08	-2.25 (0.71)	0.005	-1.28 (0.42)	0.006
		*		*				NS		NS		**		**

T1: 1 hour, T2: 24 hours, T3: 48 hours, T4: 1 week after placement of alignment archwires. *p<0.05 **p<0.01 ***p<0.001

Bivariate correlation analysis revealed no significant correlation between average PBF and pain scores after 24 hours (Pearson coefficient = -0.018, p=0.915) and 7 days (Pearson coefficient = 0.044, p=0.791) of force application.

Harms: Negative outcomes were not reported by any patients during the trial.

## Discussion

This study was aimed to investigate the existence of any damage on pulp vasculature related to the use of an 0.018-inch superelastic NiTi as the first alignment archwire during orthodontic treatment and to compare its effect with that by smaller diameter archwires. Based on the outcome of comparisons (effect on PBF), the safe use of a larger diameter 0.018-inch NiTi as first alignment archwire will be determined. The validity of using laser Doppler techniques for recording pulpal blood flow has been previously confirmed.^19^

The initial, the lag, and the post-lag are the three phases of orthodontic tooth movement. The initial phase occurs 24 hours to 48 hours after the first application of force. It is characterized by immediate and rapid movement. The lag phase is marked by PDL hyalinization in the compression area. It shows relatively minor or no tooth displacement that lasts for 20 to 30 days, and the post-lag phase, during which the rate of tooth movement increases.^3^ In our study, PBF was recorded in the aforementioned time points to keep in line with the cellular and vascular tissue changes that occur in response to orthodontic force and to enable comparison with previous studies that measured PBF at these time point intervals.^10,12-17^

The joining of the 2 different-sized NiTi archwires by crimpable hook in the midline would enable comparison of the 2 archwire sizes in the same patient, however, this may affect the physical properties of the archwires. Perumalla, et al.^20^ (2019) reported that load deflection of 0.016NiTi archwire was the highest when the length of active archwire was reduced. Also, Whitley and Kusy^21^ (2007) found that frictional resistance increases with reduced inter attachment span. As the crimpable hook is inserted, the interbracket span between the 2 lower central incisors and the length of the active NiTi archwire will be reduced. However, in our study, none of the cases showed increased antero-posterior displacement between the two lower central incisors contact area which indicates none of the aforementioned two factors was significant.

The use of rubber dam during PBF measurements with LDF has been recommended by previous studies. Researchers have suggested that it not only acts as an optical barrier, but also applies pressure to the gingival region, decreasing the regional blood flow.^13,22-23^ In our study, a rubber dam was not used, since the resulting compression is difficult to control when a fixed orthodontic appliance is present. A well-adapted silicone splint, utility wax covering the gingival margins and cotton rolls were used in to reduce the contamination from the gingival regions and act as optical barrier.^18^

In our study, a reduction in PBF 20 minutes after the insertion of alignment archwires irrespective of their size. The greatest reduction in PBF was observed at 24 hours and continued with a slower rate until it reached its maximum at 72 hours of archwires placement. This may be explained by pulpal vascular changes that accompanies orthodontic force application (the constriction of vessels that enter and leave the apical foramen) and the increased interstitial pressure in pulp chamber due to the inflammatory reaction in periodontal tissues, which results in a vascular compression, thus reducing blood flow.^24^ These findings agreed with some studies,^10, 13-14,17^but disagreed with others.^11,12,16^

Abu Alhaija, et al.^10^ (2019) and Abu Alhaija and Taha^17^ (2021) reported a similar PBF decrease during alignment and leveling after orthodontic application using clear aligners, conventional and self-ligating fixed appliances for the aforementioned time points intervals. On the other hand, Barwick and Ramsay^11^(1996) evaluated the effect of a 4-minute application of intrusive orthodontic force and suggested that PBF was unaltered. The short time of force application in their study may explain the difference in their finding compared to that of our study. Also, McDonald and Pitt Ford^12^ (1994) reported an initial decrease in PBF that lasted for 32 minutes, after which it increased to above the normal baseline values for 48 hours before returning to normal at 72 hours. In their study,^12^ they used stainless steel palatal finger spring to retract the canines, therefore, higher intermittent forces that only lasted for approximately 30 minutes were produced. In our study, superelastic NiTi archwires which deliver light contentious forces were used for alignment which may explain the difference in the results obtained. Furthermore, Sabuncuoglu and Ersahan^16^ (2014) reported that PBF reduced significantly 3 days after intrusive force application. In their study, the intrusive force was applied on upper incisors using NiTi coil spring from 0.016X0.X022-inch base archwire to a mini-implant, whereas we used contentious archwire for alignment, resulting in different types of tooth movements, not only intrusion. Differences in stress distribution related to different force application and different types of tooth movements may have contributed to the different PBF changes between the study of ours and of Sabuncuoglus and Ersahans^16^.

The lack of any PBF differences between 0.018-inch and the other smaller sized NiTi archwires could be explained by the different amount of teeth displacement which ranged from 0.5 mm in some teeth to 2.0 mm in the others. The amount of force delivered by 0.018-inch NiTi archwire ranged from 158.85 N at 0.5 mm deflection to 400.40 N at 2.0 mm deflection. This force range overlapped the amount of force delivered by the 0.016-inch NiTi archwire at 1 mm and 2 mm displacements and was close to that delivered by 0.014-inch NiTi archwire at 1 mm deflection.^6^

The general PBF decrease was comparable among all archwire sizes. After 1 week of force application, PBF started to increase and reverted to its normal values within 1 month. This is probably due to the decay in orthodontic forces with the subsequent fading of inflammatory tissue reaction, which normally occurs after orthodontic force application.

This finding was in agreement with results from other studies.^13,16-17^Salles, et al.^14^ (2013) reported no difference between blood-flow signals on day 30 and the corresponding basal values using 12 maxillary left central incisors during alignment and leveling (super-elastic wire 0.014-inch) using LDF. Sabuncuoglu and Ersahan^16^ (2014) concluded that PBF values tend to return to their initial levels after 3 weeks in both light (40 g) and heavy (120 g) intrusive force groups. These findings stress the importance of leaving a period of one month between activations to allow for the PBF to return to normal and minimize possible subsequent side effects. Abu Alhaija and Taha^17^(2021) evaluated PBF changes in conventional and self-ligating fixed orthodontic brackets using 0.016×0.022 NiTi for alignment. They found that in both groups, PBF started to increase 1 week after archwire placement, until it reached its normal values 1 month later.

Scott, et al.^25^ (2008) evaluated the effect of different appliances on pain experience. In a randomized clinical trial, they25 found that the highest discomfort occurs between 4 and 24 hours following initial archwire insertion, which diminishes by day 3 and is at a minimal baseline level by 7 days.

Little correlation exists between the degree of pain response and applied force magnitude.^26^ In our study, the perceived pain scores of the three different size archwires were similar. This finding implies that orthodontic treatment will elicit a painful response regardless of archwire size, agreeing with the results of Erdinç and Dinçer^27^ (2004). Luppanapornlarp, et al.^9^ (2010), in turn, suggested that the mean VAS score of pain intensity from higher force was significantly greater than from mild force at 24 hours.

The concept of mild forces creating more physiological and less painful tooth movement is controversial as the optimal force might differ for each tooth and for each patient.^3^ Jones and Richmond^26^ (1985) evaluated the relationship between initial tooth positions, applied force levels, and experienced pain but observed no statistically significant correlation among the three parameters. Each patient experiences pain differently.

In our study, although PBF reduction within the first 24 hours of force application was accompanied by increase in pain scores, this association was insignificant. Yu, et al.^28^ (2002) showed that the microvascular tone is modulated locally to match the tissue demands as the PBF reduces. The variability of pulp tissue response among individuals to the application of orthodontic force may explain the variability in pain experience by the patients and hence, the lack of any association.

The split-mouth design used in our study ensures that pain experienced is not due to biological differences between the patients, but due to force application related to archwire sizes and the amount of archwire deflection.

Despite the signiﬁcant short-term regressive changes in pulpal tissue during initial alignment stage, blood vessel function was maintained throughout, as indicated by PBF changes during treatment. Our findings suggest that PDF changes related to the use of superelastic 0.018-inch NiTi archwire for initial alignment does not result in irreversible changes in pulp vasculature. The PBF changes produced by 0.018-inch NiTi is similar to those resulting from 0.014-inch NiTi and 0.016-inch NiTi; indicating that, although they produce higher forces, they still are within the limit of optimal force required for orthodontic tooth movement.

As limitations, we cite the fact that PBF was measured on different teeth experiencing different types of tooth movements, alignment archwire deflection was not the same on every tooth, which might have affected the amount of force delivered by each archwire/tooth. Moreover, the presence of orthodontic appliance, which limits the measurement area. Finally, the use of silicone splint for probe stabilization and cotton rolls for surrounding tissue isolation has been proven not enough.^29^ The recommended use of combined rubber dam and rigid splint during PBF measurements was not possible in our study. The presence of orthodontic brackets attached to examined teeth made the use of rubber dam for tissue isolation impractical. Besides, PBF was measured during active orthodontic treatment, that is, the teeth were not in a fixed position. The presence of brackets, archwires, and non-stationary teeth, precluded the use of custom fabricated rigid splint for each tooth as recommended by Sabuncuoglu and Ersahan.^15^

### Generalizability

Orthodontic clinicians can use 0.018-inch NiTi as first alignment archwire with no irreversible changes to pulp vasculature.

## Conclusions

The use of 0.018-inch superelastic NiTi as the first alignment archwire does not cause irreversible PBF changes neither cause more pain.

PBF changes were similar among alignment NiTi archwires regardless of their sizes.

PBF decreased immediately after archwire placement. Most PBF changes occurred within 24 hours, continued to decrease until 72 hours, started to recover afterwards and returned to its normal values within 1 month.

Pain scores perceived by patients were similar for the 3 alignment archwires.

**Table 2:** Diff. between means and p-values for the PBF from baseline (T0) to (T5) for each measured tooth in 0.014-inch NiTi archwire

Tooth	Diff	Diff	Diff	Diff	Diff	Diff
	T0-T1	T1-T2	T2-T3	T3-T4	T4-T5	T0-T5
First Molar	0.93	1.75	1.38	-2.04	-1.99	0.04
	***	***	***	***	***	
2^nd^ premolar	0.92	1.66	1.45	-1.99	-1.96	0.09
	***	***	***	***	***	
1^st^ premolar	0.91	1.92	1.48	-2.11	-2.07	0.13
	***	***	***	***	***	
Canine	0.95	1.8	1.48	-2.05	-2.22	-0.04
	***	***	***	***	***	
Lateral incisor	0.98	1.72	1.51	-2.06	-1.96	0.19
	***	***	***	***	***	
Central incisor	0.9	1.75	1.52	-2.07	-1.95	0.15
	***	***	***	***	***	

*p<0.05 **p<0.01 ***p<0.001

**Table 3:** Diff. between means and p-values for the PBF from baseline (T0) to (T5) for each measured tooth in 0.016-inch NiTi archwire

Tooth	Diff	Diff	Diff	Diff	Diff	Diff	Diff
	T0-T1	T1-T2	T2-T3	T3-T4	T4-T5	T4-T5	T0-T5
First Molar	0.87	1.97	1.43	-1.87	-1.98	-1.98	0.42
	***	***	***	***	***	***	
2^nd^ premolar	0.82	1.92	1.36	-1.8	-1.78	-1.78	0.52
	***	***	***	***	***	***	
1^st^ premolar	0.97	1.61	1.49	-1.78	-1.95	-1.95	0.34
	***	***	***	***	***	***	
Canine	0.98	1.77	1.49	-1.9	-1.9	-1.9	-0.45
	***	***	***	***	***	***	*
Lateral incisor	0.94	1.78	1.31	-1.64	-1.95	-1.95	0.44
	***	***	***	***	***	***	
Central incisor	0.73	1.93	1.54	-1.8	-1.85	-1.85	0.55
	***	***	***	***	***	***	*

*p<0.05 **p<0.01 ***p<0.001

**Table 4:** Diff. between means and p-values for the PBF from baseline (T0) to (T5) for each measured tooth in 0.018-inch NiTi archwire

Tooth	Diff	Diff	Diff	Diff	Diff	Diff
	T0-T1	T1-T2	T2-T3	T3-T4	T4-T5	T0-T5
First Molar	.0.93	1.79 ***	1.49	-1.83	-1.88	0.5
	***		**	***	***	**
2^nd^ premolar	0.86	1.77	1.45	-1.82	-1.77	0.49
	***	***	***	***	***	***
1^st^ premolar	0.99	1.59	1.56	-1.82	-1.88	0.43
	***	***	***	***	***	**
Canine	0.99	1.72	1.47	-1.86	-1.84	0.48
	***	***	***	***	***	***
Lateral incisor	0.98	1.64	1.45	-1.76	-1.88	0.42
	***	***	***	***	***	***
Central incisor	0.84	1. 75	1.57	-1.79	-1.86	0.5
	***	***	***	***	***	***

**p<0.01 ***p<0.001
